# Supplementary material for: Polyketide synthase gene clusters in the ericoid mycorrhizal fungus Oidiodendron maius: genome-wide prediction, expression profiling, and functional characterization of knockout mutants
Source: Front Plant Sci. 2026 Sep 16;17:1924430. doi: 10.3389/fpls.2026.1924430 (PMC13623903; doi:10.3389/fpls.2026.1924430)
Supplement: Supplementary file 1 [file SupplementaryFile1.zip › Supplementary Materials/Supplementary Folder 1.zip!/Oidma1_AssemblyScaffolds_Repeatmasked/knownclusterblast/cluster41/jgi.p_Oidma1_44992_mibig_hits.html]

| MIBiG Protein | Description | MIBiG Cluster | MiBiG Product | % ID | % Coverage | BLAST Score | E-value |
| --- | --- | --- | --- | --- | --- | --- | --- |
| BAC20568.1 | efflux\_pump | BGC0000039 | Compactin\_biosynthetic\_gene\_cluster | 49 | 95.5035971223 | 497 | 2.8e-140 |
| ABA02247.1 | efflux\_pump | BGC0000098 | Monacolin\_K\_biosynthetic\_gene\_cluster | 49 | 95.3237410072 | 486 | 3.7e-137 |
| AAD34558.1 | unknown | BGC0000089 | Lovastatin\_biosynthetic\_gene\_cluster | 47 | 97.4820143885 | 480 | 3.5e-135 |
| ADY16699.1 | TqaJ | BGC0001142 | Tryptoquialanine\_biosynthetic\_gene\_cluster | 39 | 93.7050359712 | 385 | 9e-107 |
| CAP96439.1 |  | BGC0000420 | Roquefortine\_C\_/\_meleagrin\_biosynthetic\_gene\_cluster | 39 | 88.6690647482 | 350 | 4.2e-96 |
| BAD29973.1 | transporter\_protein | BGC0000676 | Aphidicolin\_biosynthetic\_gene\_cluster | 39 | 90.2877697842 | 347 | 2.7e-95 |
| AGN71625.1 | putative\_HC-toxin\_efflux\_carrier\_TOXA | BGC0000027 | Azaphilone\_biosynthetic\_gene\_cluster | 53 | 58.4532374101 | 342 | 6.7e-94 |
| CBF87265.1 | MFS\_toxin\_efflux\_pump\_(AflT),\_putative\_(AFU\_orthologue;\_AFUA\_1G12620) | BGC0000680\_c2 | Austinol/dehydroaustinol,\_locus\_1\_biosynthetic\_gene\_cluster | 39 | 88.6690647482 | 337 | 2.8e-92 |
| AAS90069.1 | AflT | BGC0000010 | Aflatoxin\_biosynthetic\_gene\_cluster | 36 | 98.9208633094 | 336 | 4.8e-92 |
| EAQ86390.1 | hypothetical\_protein | BGC0001405 | Chaetoviridin\_/\_chaetomugilin\_biosynthetic\_gene\_cluster | 41 | 78.0575539568 | 336 | 4.8e-92 |
| XP\_002384861.1 | MFS\_multidrug\_transporter,\_putative | BGC0001234\_c2 | 4,4'-piperazine-2,5-diyldimethyl-bis-phenol\_biosynthetic\_gene | 37 | 85.6115107914 | 336 | 8.2e-92 |
| ACD39765.1 | major\_facilitator\_superfamily\_transporter | BGC0000077 | Hypothemycin\_biosynthetic\_gene\_cluster | 38 | 94.6043165468 | 335 | 1.1e-91 |
| ACD39756.1 | major\_facilitator\_superfamily\_transporter | BGC0000076 | Hypothemycin\_biosynthetic\_gene\_cluster | 38 | 94.6043165468 | 335 | 1.1e-91 |
| AMR44276.1 | MFS\_multidrug\_transporter | BGC0001398\_c3 | Phomopsins\_biosynthetic\_gene\_cluster | 37 | 94.964028777 | 332 | 9e-91 |
| AAS90046.1 | AflT | BGC0000009 | Aflatoxin\_biosynthetic\_gene\_cluster | 37 | 92.0863309353 | 330 | 3.4e-90 |
| AAS90021.1 | AflT | BGC0000008 | Aflatoxin\_biosynthetic\_gene\_cluster | 36 | 98.9208633094 | 327 | 2.2e-89 |
| AAS90092.1 | AflT | BGC0000006 | Aflatoxin\_biosynthetic\_gene\_cluster | 36 | 98.9208633094 | 327 | 2.2e-89 |
| AAS89998.1 | AflT | BGC0000007 | Aflatoxin\_biosynthetic\_gene\_cluster | 37 | 91.5467625899 | 327 | 2.2e-89 |
| BAE71313.1 | putative\_ABC\_transporter | BGC0000004 | Aflatoxin\_biosynthetic\_gene\_cluster | 38 | 80.7553956835 | 312 | 1.3e-84 |
| CCE28984.1 | probable\_DHA14-like\_major\_facilitator;\_ABC\_transporter | BGC0001365 | Clapurines\_biosynthetic\_gene\_cluster | 33 | 97.8417266187 | 304 | 2e-82 |
| AGC95323.1 | CurE | BGC0000045 | Dehydrocurvularin\_biosynthetic\_gene\_cluster | 35 | 87.4100719424 | 298 | 1.9e-80 |
| AHV78251.1 | ResE | BGC0001246 | Trans-resorcylide\_biosynthetic\_gene\_cluster | 35 | 89.2086330935 | 291 | 2.3e-78 |
| ADM34144.1 | efflux\_pump | BGC0001084 | Notoamide\_/\_stephacidin\_biosynthetic\_gene\_cluster | 35 | 90.4676258993 | 287 | 2.5e-77 |
| ADI24949.1 | GsfJ | BGC0000070 | Griseofulvin\_biosynthetic\_gene\_cluster | 36 | 90.1079136691 | 277 | 2.6e-74 |
| CBF76046.1 | conserved\_hypothetical\_protein | BGC0001399 | Fellutamide\_B\_biosynthetic\_gene\_cluster | 32 | 83.8129496403 | 261 | 2.6e-69 |
| EAA65599.1 | hypothetical\_protein | BGC0000022 | Asperfuranone\_biosynthetic\_gene\_cluster | 38 | 72.4820143885 | 259 | 9.7e-69 |
| ctg1\_orf0002 |  | BGC0000688 | Copalyl\_diphosphate\_biosynthetic\_gene\_cluster | 35 | 75.1798561151 | 228 | 1.8e-59 |
| CAF60521.1 | putative\_efflux\_protein | BGC0000704 | Kanamycin\_biosynthetic\_gene\_cluster | 31 | 87.7697841727 | 207 | 3.4e-53 |
| CAF31575.1 | putative\_kanamycin\_efflux\_protein | BGC0000705 | Kanamycin\_biosynthetic\_gene\_cluster | 31 | 87.7697841727 | 207 | 3.4e-53 |
| ACS68556.1 | major\_facilitator\_superfamily\_protein | BGC0001026 | NG-391\_biosynthetic\_gene\_cluster | 30 | 88.6690647482 | 201 | 2.4e-51 |
| EAU36749.1 | predicted\_protein | BGC0000292 | Acetylaranotin\_biosynthetic\_gene\_cluster | 36 | 61.690647482 | 201 | 3.1e-51 |
| AMY15055.1 | MFS\_transporter | BGC0001339 | Squalestatin\_S1\_biosynthetic\_gene\_cluster | 30 | 82.9136690647 | 199 | 9.1e-51 |
| BAE56594.1 |  | BGC0001123 | Aspirochlorine\_biosynthetic\_gene\_cluster | 29 | 90.2877697842 | 192 | 1.1e-48 |
| AAF00219.1 | transporter | BGC0000277 | Urdamycin\_biosynthetic\_gene\_cluster | 28 | 90.1079136691 | 192 | 1.1e-48 |
| AHW57792.1 | PgaJ3 | BGC0000262 | Rabelomycin\_biosynthetic\_gene\_cluster | 27 | 85.2517985612 | 177 | 4.9e-44 |
| ACP19369.1 | SaqJ1 | BGC0000267 | Saquayamycin\_Z\_/\_galtamycin\_B\_biosynthetic\_gene\_cluster | 29 | 87.9496402878 | 173 | 9.1e-43 |
| AGA37273.1 | efflux\_pump | BGC0000819 | Paraherquamide\_biosynthetic\_gene\_cluster | 40 | 34.5323741007 | 127 | 5.8e-29 |
| AAR98561.1 | GntO | BGC0000697 | Gentamicin\_biosynthetic\_gene\_cluster | 24 | 70.6834532374 | 99 | 9.8e-21 |
| CAF31444.1 | putative\_gentamicin\_exporter | BGC0000696 | Gentamicin\_biosynthetic\_gene\_cluster | 24 | 70.6834532374 | 99 | 9.8e-21 |
| CAF34033.1 | putative\_transmembrane\_efflux\_protein | BGC0000689 | 2-deoxystreptamine\_biosynthetic\_gene\_cluster | 24 | 70.6834532374 | 99 | 9.8e-21 |
| ABI22121.1 | transmembrane\_efflux\_protein | BGC0000422 | Saframycin\_A\_biosynthetic\_gene\_cluster | 25 | 75.7194244604 | 94 | 3.2e-19 |
| ACN38355.1 | putative\_transmembrane\_efflux\_protein | BGC0000714 | Sisomicin\_biosynthetic\_gene\_cluster | 24 | 76.0791366906 | 94 | 5.4e-19 |
| ABP55162.1 | major\_facilitator\_superfamily\_MFS\_1 | BGC0000150 | Sporolide\_biosynthetic\_gene\_cluster | 23 | 91.0071942446 | 69 | 1.9e-11 |
